# Beyond the Overlap: Understanding the Empirical Association Between ADHD Symptoms and Executive Function Impairments in Questionnaire-Based Assessments

**DOI:** 10.3390/children12080970

**Published:** 2025-07-24

**Authors:** Claudia Ceruti, Gian Marco Marzocchi

**Affiliations:** 1 Department of Psychology, University of Milan-Bicocca, 20126 Milan, Italy; c.ceruti9@campus.unimib.it; 2 Centro per l’Età Evolutiva, 24121 Bergamo, Italy

**Keywords:** Conners 3, executive function questionnaires, ADHD, executive functions, multi-informant assessment

## Abstract

**Background/Objectives**: Executive function (EF) difficulties are increasingly recognized as closely linked to ADHD, particularly when assessed via rating scales. **Methods**: The present study investigated the nature of these associations, using the Conners 3 Rating Scales to assess ADHD symptoms and the Executive Function Questionnaire (EFQU) to assess EF impairments, in a sample of 1068 children (40.8% males, 38.8% females) aged 7–14 years (M = 10.7, SD = 1.74). **Results**: Both parent and teacher ratings revealed strong correlations, particularly between inattentive symptoms and EF difficulties, across multiple executive domains. To examine whether these associations stemmed from construct or phrasing overlap, exploratory and confirmatory factor analyses were conducted. The results demonstrate that the Conners 3 and the EFQU capture distinct latent dimensions of functioning, with virtually no overlap in item content. **Conclusions**: The strength and consistency of the associations between these latent factors support the interpretation that, although conceptually distinct, ADHD symptoms and EF impairments are empirically intertwined in everyday functioning, as consistently reported by both parents and teachers. Interestingly, teachers provided more integrated views of behavior, while parents tended to distinguish ADHD and EF traits more clearly. These findings underscore the importance of multi-informant assessment and contextual variability in understanding children’s functioning.

## 1. Introduction

Hetero-report tools are widely employed as a fundamental component in the assessment of neurodevelopmental conditions, serving to collect accurate information from parents and teachers about children’s behaviors and functioning. Although individuals, including children, are uniquely positioned to report on their own experiences [1], their limited language comprehension and the reduced reliability of survey responses from young respondents compared to adults [2] make the inclusion of hetero-report tools essential during the assessment process. Nevertheless, it remains of utmost importance to gather perspectives from multiple informants in order to achieve a broader and more nuanced understanding of the child’s condition.

Questionnaires represent a fast and effective way to collect information, and they are extensively administered during the assessment of different neurodevelopmental conditions, such as the well-known Conners 3 Rating Scales [3] for Attention-Deficit/Hyperactivity Disorder (ADHD) symptoms. Two main versions of the Conners Rating Scales are offered, one for completion by parents (Conners 3-P) and one for completion by teachers (Conners 3-T), and they have been proven to be reliable and valid tools to assess ADHD. They include multiple scales for different behaviors, such as Peer Relations, Learning Problems, and Aggression, as well as Inattention and Hyperactivity/Impulsivity, which are particularly relevant to the present investigation. Recent validation studies on the Italian version of the Conners 3-P and 3-T scales have confirmed their factorial structure and internal consistency, supporting their use in both clinical and research settings. The modest correspondence that is typically observed between informants further highlights the value of adopting a multi-informant approach, as each rater contributes unique, context-specific information [4]. According to the fifth edition of the DSM [5], ADHD is a neurodevelopmental condition that is primarily characterized by symptoms in two behavioral domains: inattention and/or hyperactivity–impulsivity. Symptoms of ADHD must be present in at least two different settings, exceeding developmentally appropriate levels and causing significant impairment in social, academic, or family functioning; therefore, multi-informant assessments become essential to fully define children’s behavior and symptoms [6,7,8]. Moreover, ADHD has been increasingly recognized as a highly heterogeneous disorder, as indicated by its diversity of comorbidities, varied clinical profiles, developmental trajectories, and patterns of neurocognitive impairment [9]. Importantly, neurocognitive impairments are hypothesized to be a core component of ADHD symptomatology, particularly affecting domains of sustained attention or vigilance, self-regulation, and executive function (EF) [10,11].

EF is defined as a high-level cognitive mechanism that allows individuals to provide adequate responses to everyday life’s demands by regulating actions through goal-directed behavior [12]. It is a broad umbrella term that encompasses various abilities, including flexibility of thinking, inhibition, problem-solving, planning, impulse control, concept formation, abstract thinking, and creativity [13]. Several models have been proposed in the literature to explain the nature of executive functioning. Unitary models describe EF as a single cognitive system or resource responsible for top-down control, e.g., [14]. Sequential models view EF as a series of developmental stages or processes, e.g., [15]. However, the most widely adopted approach in developmental research is the multicomponent model, which conceptualizes EF as composed of distinct but interrelated abilities, e.g., [16,17]. This perspective informed the present study, which considers executive functioning as a heterogeneous construct, comprising multiple dimensions that may vary across individuals and developmental contexts.

Kofler and colleagues [18] demonstrated that among a sample of 136 children with ADHD, 89% displayed impairments in at least one EF domain (62% exhibited impaired working memory (WM), 27% impaired inhibitory control, and 38% impaired set shifting). Indeed, Brown [19] described ADHD as fundamentally a cognitive condition characterized by a developmental disruption in EF. Such impairments often lead to persistent challenges in managing attention, shifting focus, regulating alertness and effort, processing information efficiently, and modulating emotional responses and frustration [19,20]. Additionally, Barkley [21] proposed a widely recognized and historically influential theory of ADHD, which identifies executive dysfunction—Particularly deficits in behavioral inhibition—as the central mechanism underlying the disorder. Willcutt et al. [22] supported this perspective with a meta-analysis showing that children with ADHD consistently perform worse than typically developing peers in tasks measuring response inhibition, WM, and set shifting. However, the authors did not conceptualize ADHD as an executive disorder per se; they argued that EF deficits are neither necessary nor sufficient to account for all cases of ADHD. Instead, EF impairments should be viewed as a significant but not exclusive component of the disorder’s complex neuropsychological profile. This has been supported by a wide and robust body of literature, e.g., [23,24,25,26].

Beyond identifying general EF weaknesses, a growing line of research has explored whether specific executive domains are more closely associated with particular symptom dimensions of ADHD. Findings suggest that difficulties in WM, planning, and sustained attention are more robustly linked to inattention symptoms than to hyperactivity or impulsivity [21,22]. In their meta-analysis, Willcutt et al. [22] found that inattention showed stronger and more consistent correlations with EF task performance across studies, particularly in the domains of WM and set shifting. Building on this distinction, it becomes equally important to consider whether these associations between EF and ADHD symptoms are consistently observed across different informants and contexts. While numerous studies have examined the relationship between EF impairments and ADHD symptomatology, far fewer have explored whether this relationship is perceived consistently across different informants and observational contexts. The extent to which parents and teachers agree on the presence and severity of EF difficulties—and, crucially, on their association with ADHD symptoms—remains an understudied but clinically relevant question. Andersen et al. [27] reported that, although teachers tended to rate higher levels of EF difficulties than parents, there was a medium-to-high correlation between teacher-reported EF deficits and parent-reported functional impairment. Nonetheless, correlations between EF and ADHD symptom measures rated by different informants were less consistent, underscoring the importance of considering contextual variability in both symptom expression and functional impact. These findings align with previous work [6]. Additionally, De Los Reyes et al. [6] highlighted that discrepancies across informants may reflect not only error variance, but also meaningful differences related to source variance (i.e., informant-related characteristics), setting variance (i.e., the context in which behavior is observed), and the specific dimensions assessed. These sources of variance must be taken into account when interpreting multi-informant data, as they can shape both the magnitude and the pattern of observed associations. However, when different informants evaluate similar constructs across contexts, correspondence may still emerge, particularly when the constructs are behaviorally salient and consistently observed across settings. This framework supports the interpretation that, despite expected mean-level differences, a similar pattern of correspondence between EF and ADHD symptoms may emerge across parent and teacher ratings.

Therefore, if EF impairments are considered one of the significant features of ADHD, and given the current literature emphasizing the need to extend assessment beyond basic symptomatology [28], the evaluation of EF becomes crucial in the clinical assessment of ADHD. Despite its recognized utility in identifying specific neurocognitive impairments in children with ADHD, different EF assessment tools often yield inconsistent results. Indeed, Barkley [29] and Brown [30] have argued that EF impairments in ADHD are more effectively and validly captured through questionnaires, which assess individuals’ ability to manage everyday demands, rather than their optimal performance under controlled conditions. This concern is supported by evidence showing that only a subset of individuals with ADHD—approximately 30%—exhibit significant impairments in traditional neuropsychological EF tasks [30], raising doubts about whether such measures are sufficiently sensitive to detect the executive difficulties that are commonly observed in real-world functioning. Performance-based tasks often isolate specific cognitive processes and may fail to assess the integration of executive abilities required in complex, everyday situations [25]. This pattern not only emerged in earlier syntheses [31], but has also been confirmed by recent meta-analytic evidence [32] showing that questionnaires yield significantly larger effect sizes than performance-based measures and discriminate better between ADHD and non-ADHD groups. Converging evidence supports the conclusion that performance-based and rating measures of EF assess different constructs or expressions of EF [31,32,33,34]. While EF rating scales offer increased ecological validity and are well suited for capturing real-world functioning, it is important to note that their interpretation—like that of neuropsychological tests [35]—can be challenging due to construct impurity [31,34,36]. No available measure provides a “pure” assessment of executive functioning, as EF tasks systematically involve non-executive cognitive processes. Executive functions operate by engaging other domain-general or domain-specific abilities, introducing consistent variance related to non-EF factors that complicates the measurement of the target construct.

In the Italian context, the EFQU [37] is a hetero-report questionnaire developed to assess executive functioning in everyday settings. Rather than conceptualizing EF as a unitary ability, the instrument adopts a multidimensional perspective, viewing EF as a set of interrelated but distinct abilities. This theoretical orientation is reflected in the separate subscales for parents and teachers, which map onto different domains, such as Cognitive Self-Regulation, Behavioral Self-Control, Organization, Adaptability, and Initiative. Importantly, children with ADHD often exhibit generalized executive impairments—spanning both cognitive and emotional–behavioral domains—which are consistently observed across home and school contexts. Therefore, assessing EF through multidimensional and context-sensitive tools like the EFQU is particularly valuable. The instrument allows for the identification of specific executive profiles across different settings, highlighting convergences and divergences in functioning. This approach aligns with the broader need for multi-informant and ecologically valid assessment practices in ADHD, where executive abilities may manifest differently depending on environmental demands and relational dynamics.

### Aims and Hypotheses

The present study aims to further examine the association between ADHD symptom rating scales (e.g., Conners 3) and EF impairment scales (e.g., Executive Function Questionnaire—EFQU), investigating whether executive impairments are more closely linked to one of the two core ADHD symptom domains—inattention or hyperactivity–impulsivity—and whether EF difficulties differ depending on the context in which the child is observed—at home (parent ratings) or at school (teacher ratings). According to the previous literature, we expected to find robust correlations between ADHD symptom rating scales and EF impairment scales, particularly between the Inattention subscale of the Conners and the Cognitive Self-Regulation scale of the EFQU, across both parent and teacher ratings. Additionally, we hypothesized that EF impairments would be more strongly associated with inattention symptoms than with hyperactivity–impulsivity. We also expected a similar degree of correspondence between parent and teacher ratings, suggesting cross-informant consistency in the association between ADHD symptoms and EF difficulties.

Once these associations have been assessed, it becomes crucial to question why such strong correspondences emerge when comparing questionnaire-based assessments of ADHD symptoms and EF difficulties. Several explanations are possible:

1. First, there may be an overlap in the constructs measured by ADHD symptom rating scales (e.g., Conners 3) and EF impairment scales (e.g., EFQU);
2. Second, the similarity in item phrasing and focus between the two types of questionnaires might contribute to inflating the observed correlations;
3. Lastly, ADHD symptoms and EF difficulties, though conceptually distinct, may co-occur to such an extent in real-world behavior that they show substantial convergence in informant-based assessments.

The specific reasons underlying the strong association between ADHD and EF rating scales have not yet been thoroughly investigated. Based on prior evidence concerning ADHD and EF, we hypothesize that this correlation reflects a convergence between conceptually distinct but behaviorally overlapping domains. To support this interpretation, the present study aims to test and challenge alternative explanations, including potential construct overlap and shared item content.

## 2. Materials and Methods

### 2.1. Participants

Participants were recruited on a voluntary basis from both public and private elementary and lower secondary schools located in four Italian regions: Lombardy, Liguria, Campania, and Emilia-Romagna. After presenting the project to the school principals, written informed consent was obtained from the children’s parents or legal guardians. Subsequently, children were assessed using Raven’s Progressive Matrices. To exclude participants with intellectual disabilities, only children scoring above the 20th percentile on Raven’s Matrices were included in the final sample. No additional clinical exclusion criteria were applied; therefore, while participants were not selected based on neurodevelopmental conditions, the only confirmed inclusion criterion related to cognitive functioning. Parents and teachers of the selected sample were then asked to complete the Conners and QUFE questionnaires, with an estimated completion time of approximately 20–30 min. However, the time taken to return the completed questionnaires varied greatly across participants—from a few days to several weeks or even months—due to contextual and organizational factors (e.g., school schedule, parental availability) rather than the actual time required for completion.

The final sample consisted of 1068 children aged 7 to 14 years, for whom parent and/or teacher questionnaires were completed. Within this sample, 547 referred to males (40.8% of the total sample) and 520 to females (38.8%), with a mean reported age of 10.7 years (DS = 1.74). Regarding school grade distribution, 35 questionnaires (2.6%) referred to children in second grade, 204 (15.2%) in third grade, 194 (14.5%) in fourth grade, 201 (15.0%) in fifth grade, 160 (11.9%) in sixth grade (first year of middle school), 141 (10.5%) in seventh grade (second year of middle school), and 132 (9.9%) in eighth grade (third year of middle school). Additional information regarding the distribution of participants across age groups and gender can be found in Table 1. For 38 participants, precise age data were not available; only their school grade was recorded.

Information on bilingualism was available for 1067 children. Of these, 13.1% were reported to be bilingual, while the remaining 86.9% were monolingual. No additional data were collected on the specific languages spoken or the degree of language exposure. Additionally, information on parental education was collected through a brief demographic questionnaire. Among mothers (N = 941), 2.6% reported having completed only primary school or having no formal education, 13.7% had a lower secondary school diploma, 48.8% held an upper secondary school diploma, 27.5% had a university degree, and 7.4% held a postgraduate qualification (master’s or doctoral degree). Among fathers (N = 920), 2.7% had completed only primary school or had no formal education, 25.7% had a lower secondary school diploma, 47.6% held an upper secondary school diploma, 19.5% had a university degree, and 4.6% held a postgraduate qualification.

Among the 1068 children included in the study, parent- and/or teacher-completed questionnaires were available in varying proportions: 811 EFQU forms were completed by parents (75.9%) and 994 by teachers (93.1%), while 705 Conners 3 forms were completed by parents (66.0%) and 837 by teachers (78.4%). This variability was taken into account in handling missing data.

The study was conducted in accordance with the ethical standards of the Declaration of Helsinki and was approved by the Ethics Committee of the University of Genoa (protocol code 2022/16, 17 February 2022).

### 2.2. Measures

Conners 3 Rating Scales [3]. Conners 3, developed by C. Keith Conners [3], represents a comprehensive tool for assessing ADHD and related learning, behavioral, and emotional difficulties in children and adolescents. In the present report, the Italian adaptation [3] was employed, specifically the versions for teachers (115 items: 113 closed-ended and 2 open-ended questions) and parents (110 items: 108 closed-ended and 2 open-ended questions). Responses are based on the child’s or student’s behavior over the past month, and are rated on a 4-point Likert scale ranging from “0—Not true at all” to “3—Very true.” The Conners 3 assessment encompasses several subscales that evaluate key behavioral domains. Specifically, in the present study, the following subscales were considered, for both parents and teachers: Inattention (Conners—IN), assessing difficulties in maintaining attention and organization, and Hyperactivity/Impulsivity (Conners—H/I), measuring excessive activity levels and impulsive behaviors.

The Conners–IN subscale is composed of 10 items, while the Conners–H/I subscale includes 11 items; this structure applies to both the parent and teacher versions. Concerning reliability, the Conners–IN and Conners–H/I subscales showed excellent internal consistency, with Cronbach’s alpha values of 0.90 and 0.89, respectively, for the parent version, and 0.96 for both subscales in the teacher version, as reported in the Italian validation study [4].

All Conners subscales were scored using standardized T-scores, with higher scores indicating greater symptom severity. On a practical level, item-level responses, collected via paper or Google Forms, were entered into a preformatted Excel spreadsheet designed to calculate the T-scores for the Conners–IN and Conners–H/I subscales. Omitted responses were treated as missing values and excluded from scoring.

The EFQU [37]. The EFQU is a self-report questionnaire designed for parents (EFQU-P) and teachers (EFQU-T) to independently assess the executive functions of children and adolescents aged 7 to 14. It includes 32 items rated on a 5-point Likert scale, ranging from “1—Completely untrue” to “5—Completely true.” The EFQU evaluates several domains, such as cognitive skills, emotional and cognitive regulation, material management, adaptability, and initiative. The parent version yields six scores (Cognitive Self-Regulation, Behavioral Self-Control, Material Management, Adaptability, Initiative, and a Total EF score), while the teacher version provides four scores (Self-Regulation, Self-Organization, Material Management, and a Total EF score).

For the EFQU-P, the Cognitive Self-Regulation subscale includes 11 items, the Behavioral Self-Control subscale includes 9 items, the Material Management subscale includes 4 items, the Adaptability subscale includes 4 items, and the Initiative subscale includes 4 items. For the EFQU-T, the Self-Regulation and Self-Organization subscales each consist of 14 items, while the Material Management subscale includes 4 items. The internal consistency is good for both the parent (α = 0.95) and the teacher (α = 0.98) versions [37].

All EFQU subscales were scored using raw scores, with higher scores reflecting greater EF abilities. As with the Conners questionnaire, item-level responses to the EFQU, collected via paper or Google Forms, were entered into a preformatted Excel spreadsheet designed to compute the sum scores for each EFQU subscale. Omitted responses were treated as missing values and excluded from scoring.

### 2.3. Statistical Analyses

Potential outliers were examine using z-score analyses (threshold |z| > 3). Although a few statistical outliers were detected on some scales (e.g., ranging from 1 to a maximum of 4 outliers in the EFQU-P—Cognitive Self-Regulation scale), none were removed, as they represented genuine behavioral variability and meaningful individual differences within a dimensional framework. Prior to conducting the main analyses, data were screened for missing values. The percentage of missing data for each variable is reported in Table 2. To assess the randomness of missingness, Little’s MCAR (Missing Completely At Random) test was performed, yielding a non-significant result (χ^2^(110) = 56.22, *p* = 1.000), indicating that data were missing completely at random. The normality of the distributions was assessed through the Kolmogorov–Smirnov and Shapiro–Wilk tests, both of which were significant (*p* < 0.001) for all variables, indicating deviations from normality. Furthermore, several variables showed skewness and kurtosis values beyond the acceptable range (±1), confirmed by visual inspection of histograms. Descriptive statistics for all subscales, including skewness, kurtosis, and missing data, are reported in Table 2. Given these results, non-parametric analyses (Spearman’s rho) were adopted to ensure greater robustness. Spearman correlation analyses were conducted to investigate the association between ADHD symptom rating scales and EF impairment scales. Missing data were handled using listwise deletion, to ensure consistency across pairwise comparisons. In line with conventional benchmarks for meaningful effect sizes [38], only correlations with a magnitude of r ≥ 0.50 were considered and discussed.

Furthermore, exploratory factor analyses (EFAs) were conducted to examine whether the Conners 3 and the EFQU assess overlapping dimensions of behavior, in order to evaluate the extent to which the two instruments might tap into shared underlying constructs. In order to manage missing data in the item-level EFA, missing values were replaced using the mean substitution method. Subsequently, confirmatory factor analyses (CFAs) were carried out to test the factorial validity of the resulting structures. Prior to each CFA, the factorial solutions were reviewed for theoretical and conceptual coherence, and small adjustments were made. Model fit was assessed using standard indices (e.g., CFI, TLI, RMSEA, SRMR), and the reliability of each latent construct was evaluated through Cronbach’s alpha. Model fit was evaluated using commonly recommended criteria [39,40]. Specifically, Comparative Fit Index (CFI) values ≥ 0.90 were considered indicative of adequate fit, and values ≥ 0.95 indicative of excellent fit. Root Mean Square Error of Approximation (RMSEA) values ≤ 0.05 indicated good fit, values between 0.05 and 0.08 indicated acceptable fit, and values ≥ 0.10 indicated poor fit. Standardized Root Mean Square Residual (SRMR) values < 0.10 were considered indicative of acceptable model fit. Cronbach’s alpha values ≥ 0.70 were considered acceptable, those ≥0.80 were considered good, and those ≥0.90 were considered excellent [41]. The sample size was considered adequate for the planned factor analyses, as it aligned with the commonly adopted subject-to-item ratio of around 10:1 [42].

Latent factor correlations were also inspected to explore the relationships among the underlying dimensions assessed by the EFQU and the Conners 3 questionnaires. New composite variables were computed by averaging the items corresponding to each theoretical factor. Spearman’s correlations were then used to examine the associations between factors, with listwise deletion applied to handle missing data.

Data were analyzed using IBM SPSS 29.0 (SPSS, Chicago, IL, USA) and Jamovi 2.5.5 (The Jamovi Project, 2021). The alpha level was set at 0.05 for all statistical tests.

## 3. Results

### 3.1. Correlation Analyses for Questionnaire Subscales

Spearman correlation analyses indicated significant results for all associations. Concerning parents’ ratings, the Conners—IN subscale negatively correlates with the EF Total score (*r*(639) = −0.64, *p* < 0.001) and the Conners—H/I subscale also shows a negative correlation (*r*(639) = −0.45, *p* < 0.001). Similar results were found also for teachers’ ratings: the Conners—IN subscale negatively correlates with the EF Total score (*r*(822) = −0.65, *p* < 0.001), as well as the Conners—H/I (*r*(822) = −0.46, *p* < 0.001). Inattention symptoms appear to be more strongly associated with EF impairments than hyperactivity/impulsivity symptoms, for both parent and teacher ratings. No meaningful differences emerged between home and school contexts for the total EF scores.

A more detailed analysis of the EFQU-P subscales revealed significant negative correlation between the Conners—IN subscale and four EF domains: Cognitive Self-Regulation (*r*(639) = −0.65, *p* < 0.001), Behavioral Self-Control (*r*(639) = −0.47, *p* < 0.001), Material Management (*r*(639) = −0.47, *p* < 0.001), and Initiative (*r*(639) = −0.45, *p* < 0.001). In contrast, for the Conners—H/I subscale, only one significant association emerged, with Behavioral Self-Control (*r*(639) = −0.47, *p* < 0.001). For teachers’ ratings, the Conners—IN subscale showed significant negative correlations with all EF domains: Self-Regulation (*r*(822) = −0.53, *p* < 0.001), Self-Organization (*r*(822) = −0.67, *p* < 0.001), and Material Management (*r*(822) = −0.55, *p* < 0.001). The Conners—H/I subscale was significantly associated only with Self-Regulation (*r*(822) = −0.53, *p* < 0.001) and Material Management (*r*(822) = −0.42, *p* < 0.001). These results show that the Conners—IN subscale has the strongest and most consistent associations with EF difficulties, across multiple executive domains and informants. Although no substantial differences emerged between home and school contexts, teachers’ ratings appeared slightly more strongly associated with EF impairments.

### 3.2. Factor Analyses

To further investigate the overlap between the EFQU and Conners 3 items, two EFAs were conducted: one for parents’ ratings and one for teachers’ ratings. The analysis used Principal Component Analysis as the extraction method and Oblimin rotation (with Kaiser normalization) to allow for correlation among components. The Kaiser–Meyer–Olkin (KMO) measures confirmed sampling adequacy, and Bartlett’s tests of sphericity indicated that both correlation matrices were factorable (*p* < 0.001), supporting the appropriateness of conducting exploratory factor analyses for the parent and teacher datasets.

The first explanatory analysis concerned parents’ ratings, and examined the 32 items of the EFQU-P and the 21 items of the Conners—IN and—H/I rated by parents. It identified eight factors explaining 60.24% of the total variance: (I) Cognitive Self-Regulation, incorporating 11 items from the EFQU-P (1, 4, 7, 8, 11, 14, 17, 18, 20, 22, 25), explaining 32.09% of the total variance; (II) Hyperactivity, comprising eight items from the Conners—P (45, 54, 61, 69, 71, 93, 98, 99), explaining 9.04% of the total variance; (III) Adaptability, incorporating eight items from the EFQU-P (3, 6, 13, 19, 27, 28, 29, 30), explaining 5.64% of the total variance; (IV) Material Management, incorporating five items from the EFQU-P (2, 5, 12, 26, 31) and one from the Conners—P (97), explaining 3.77% of the total variance; (V) Inattention, comprising ten items from the Conners—P (2, 28, 35, 47, 68, 79, 84, 95, 101, 43), explaining 3.11% of the total variance; (VI) Interpersonal Self-Control, incorporating four items from the EFQU-P (15, 16, 21, 24), explaining 2.44% of the total variance; (VII) Impulsivity, incorporating one item from the EFQU-P (9) and three from the Conners—P (3, 61, 104), explaining 2.19% of the total variance; and (VIII) Emotional Control, comprising two items from the EFQU-P (10, 32), accounting for 1.96% of the total variance.

**Figure 1:**
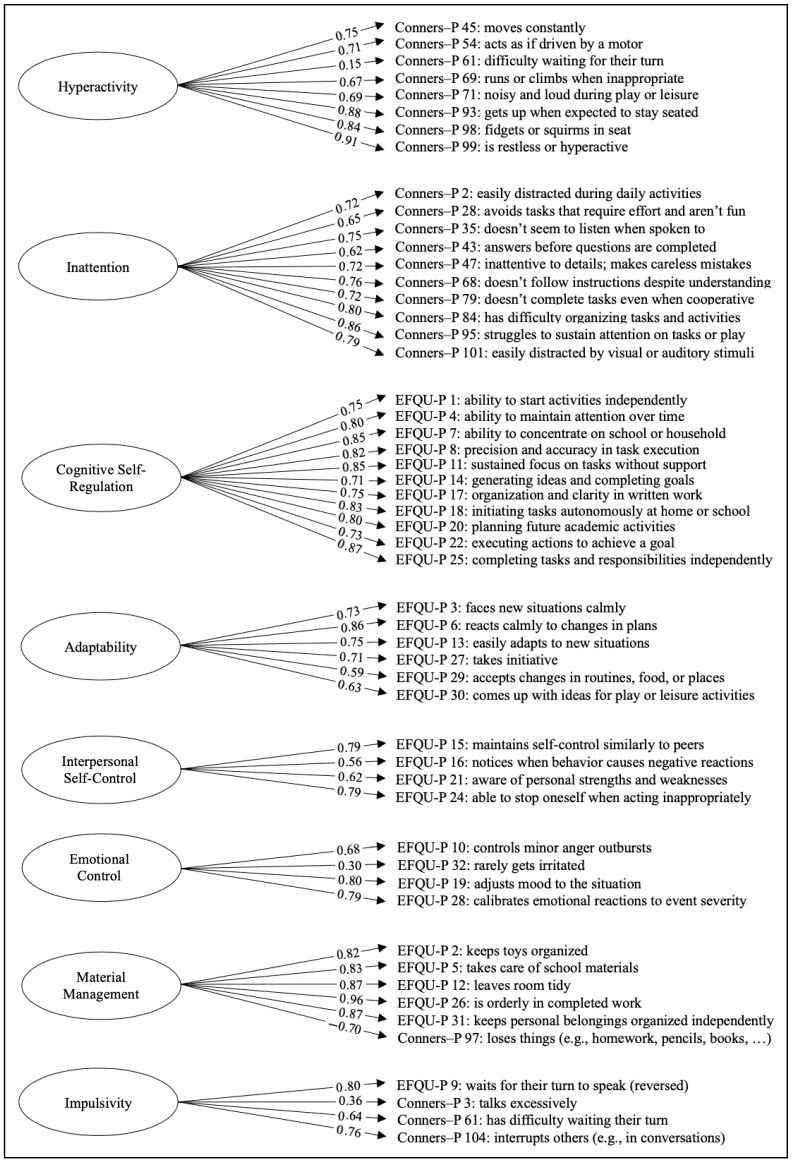
A path diagram of the confirmatory factor analysis (CFA) model based on parent ratings.

**Figure 2:**
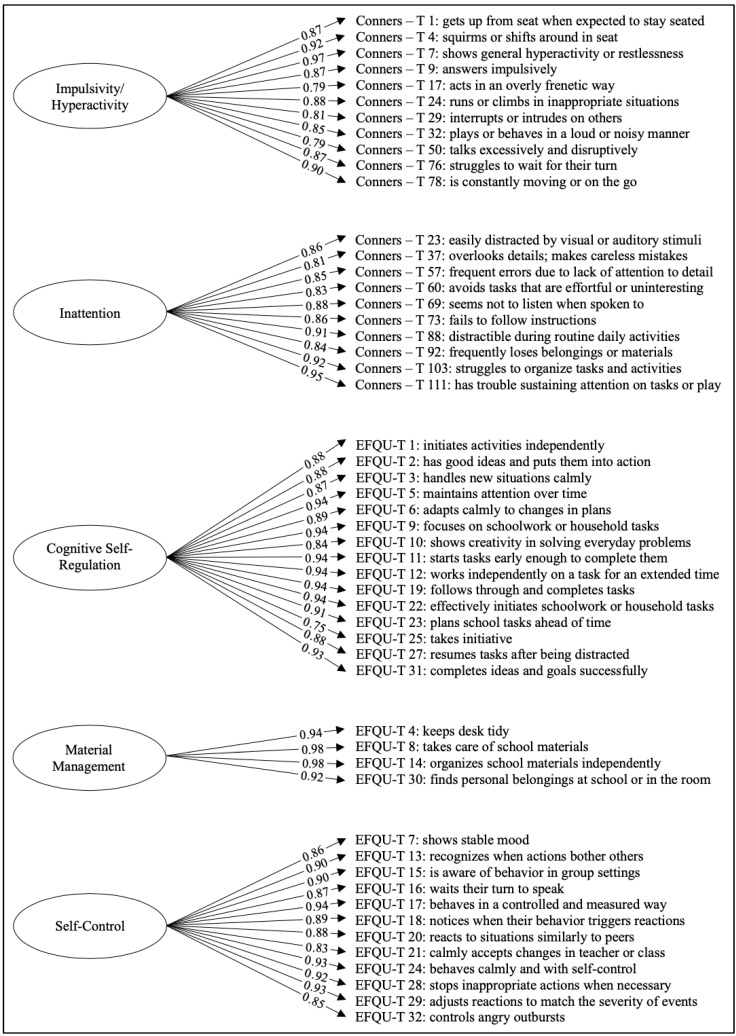
A path diagram of the confirmatory factor analysis (CFA) model based on teacher ratings.

Following the EFA, eight latent factors were identified and retained for the confirmatory analysis. Before proceeding with the CFA, the factor structure was examined conceptually to ensure theoretical coherence across constructs. To improve conceptual clarity, a refinement of the factor structure was applied. Specifically, two items, originally loading on the Adaptability factor (EFQU-P 19 and EFQU-P 28), were reassigned to the Emotional Control factor, with the aim of both clarifying the former and strengthening the latter. Notably, both reassigned items (EFQU-P 19 and EFQU-P 28) loaded strongly on the Emotional Control factor in the CFA (standardized loadings = 0.79 and 0.80, respectively), providing empirical support for the theoretical rationale behind the reassignment. Additionally, the EFQU-P 9 item was reversed to align with the directionality of the other items in the impulsivity factor. The CFA (Figure 1) was then conducted on this revised eight-factor model. The model showed a good fit to the data: χ^2^(1245) = 3927, *p* < 0.001, CFI = 0.982, TLI = 0.981, RMSEA = 0.061, SRMR = 0.062. All standardized loadings were statistically significant and above 0.30, except for one item (Conners–P 61), supporting the adequacy of the proposed structure. The low standardized loading of the Conners–P 61 item may be due to its ambiguous content, which, in the EFA, showed moderate cross-loadings on both the Hyperactivity and Impulsivity factors. Although its CFA loading was low, the item was retained within the Hyperactivity factor, given its stronger EFA saturation and theoretical alignment with the original subscale structure of the Conners Rating Scales. Additionally, to assess internal consistency, Cronbach’s alpha coefficients were computed for each scale (Table 3). All factors demonstrated acceptable to excellent reliability, with α values ranging from 0.71 (Emotional Control) to 0.94 (Cognitive Self-Regulation). For clarity, only standardized item loadings are displayed in the path diagrams (Figure 1 and Figure 2). Correlated residuals and inter-factor correlations are not shown.

The second EFA concerned teachers’ ratings, and examined the 32 items of the EFQU-T and the 21 items of the Conners—IN and —H/I rated by teachers. It identified six factors, explaining 74.33% of the total variance: (I) Cognitive Self-Regulation, incorporating fourteen items from the EFQU-T (1, 2, 3, 5, 9, 10, 11, 12, 19, 22, 23, 25, 27, 31), explaining 49.89% of the total variance; (II) Impulsivity/Hyperactivity, comprising eleven items from the Conners—T (1, 4, 7, 9, 17, 24, 29, 32, 50, 76, 78), explaining 10.86% of the total variance; (III) Inattention, incorporating ten items from the Conners—T (23, 37, 57, 60, 69, 73, 88, 92, 103, 111), explaining 6.63% of the total variance; (IV) Material Management, incorporating four items from the EFQU-T (4, 8, 14, 30), explaining 2.76% of the total variance; (V) Self-Control, comprising twelve items from the EFQU-T (7, 13, 15, 16, 17, 18, 20, 21, 24, 28, 29, 32), explaining 2.22% of the total variance; (VI) and Adaptability, incorporating one item from the EFQU-T (6), explaining 1.97% of the total variance.

Before proceeding with the CFA, the factor structure was examined conceptually to ensure theoretical coherence across constructs. To improve conceptual clarity, a refinement of the factor structure was applied: the EFQU-T 6 item, originally solely loading on the Adaptability factor, was reassigned to the Cognitive Self-Regulation factor, on which the EFQU-T 6 item had the second-highest load. Additionally, this modification was theoretically motivated, as EFQU-T 6 refers to sustained effort and goal-directed persistence—core components of cognitive self-regulation. The empirical adequacy of this choice was supported by the CFA, where the item showed a strong standardized loading on the new factor (λ = 0.89). The CFA (Figure 2) was then conducted on this revised five-factor model. The model showed a good fit to the data: χ^2^(1264) = 7343, *p* < 0.001, CFI = 0.994, TLI = 0.994, RMSEA = 0.080, SRMR = 0.066. All standardized loadings were statistically significant and above 0.30, supporting the adequacy of the proposed structure. Additionally, Cronbach’s alpha coefficients (Table 4) demonstrated excellent reliability, with α values ranging from 0.94 (Impulsivity/Hyperactivity) to 0.97 (Cognitive Self-Regulation and Self-Control).

### 3.3. Correlation Analyses for Extracted Factors

Spearman correlation analyses revealed strong and consistent associations among most latent factors identified in the parent ratings (Table 5). Notably, Cognitive Self-Regulation emerged as a central construct, showing robust associations with all EF domains, as well as with Inattention. The other EF-related variables were primarily intercorrelated among themselves. Similarly, Conners’ domains were associated with each other, except for Inattention, which also showed a strong correlation with Cognitive Self-Regulation, suggesting shared underlying regulatory processes.

Spearman correlation analyses revealed strong and consistent associations among most latent factors identified in the teacher ratings (Table 6). Cognitive Self-Regulation once again emerged as a central construct, showing strong negative associations with all other factors except Impulsivity/Hyperactivity. All remaining factors were also interrelated: both Inattention and the EF-related domains displayed significant correlations with one another. Notably, Inattention showed particularly strong links with Cognitive Self-Regulation, further supporting the overlap between attentional difficulties and executive dysfunction. Overall, these patterns suggest a coherent structure of executive functioning and behavioral regulation as observed by teachers in the classroom context.

## 4. Discussion

The first objective of the present study was to confirm the association between behavioral manifestations of ADHD and difficulties in EF. The correlation analysis demonstrated that both inattentive and hyperactive/impulsive ADHD symptoms are strongly linked to EF impairments, across both informant groups. This result fits well within a wide and robust body of literature, which consistently reports strong associations between ADHD and EF deficits [18,22,32]. Specifically, inattention symptoms showed higher associations with EF difficulties than hyperactivity/impulsivity symptoms, suggesting that inattentive behaviors are more closely tied to executive impairments. This finding aligns with the previous literature suggesting that EF deficits are more prominently linked to the inattentive dimension of ADHD than to hyperactivity/impulsivity, e.g., [21,22]. Interestingly, no meaningful differences emerged between home and school settings, indicating that the relationship between ADHD symptoms and EF impairments is robust across different environments and informants, with inattentive symptoms consistently showing stronger executive associations. Even though the literature on this specific topic is not very extensive, prior research has indeed indicated that inattentive symptoms tend to exhibit stable associations with EF difficulties across different settings and informants [27]. This result further corroborates the robust link between ADHD and EF impairments, demonstrating that this association is consistently present and strong across contexts.

A deeper analysis was conducted to further explore the associations between the Conners—IN and —H/I subscales and the EFQU domains, in order to better understand the patterns observed in the total scores. Parents rated children with inattentive symptoms as particularly struggling to autonomously initiate, sustain, and complete tasks, reflecting core aspects of organization, planning, and goal-directed behavior (Cognitive Self-Regulation). These children were also described as being less able to inhibit impulsive or inappropriate responses and to regulate emotional reactions (Behavioral Self-Control), as well as being less capable of maintaining order and taking care of personal belongings (Material Management), and, ultimately, of taking initiative, generating ideas, and demonstrating awareness of personal strengths (Initiative). In contrast, parents associated hyperactive and impulsive symptoms only with difficulties in regulating emotional reactions and maintaining socially appropriate behavior (Behavioral Self-Control). A similar pattern was observed in the teacher ratings. Inattentive symptoms, as reported in the Conners—IN subscale, showed significant associations with all EF domains assessed by the EFQU-T. Teachers rated children with high levels of inattention as primarily displaying a poorer capacity to manage impulses, remain calm, adapt to changes, and behave appropriately in social contexts (Behavioral Self-Regulation). These children were also described as less capable of sustaining attention over time, persisting after setbacks, and independently organizing their workflow (Cognitive Self-Regulation), and as having difficulties in maintaining order, keeping track of materials, and managing their physical environment (Material Management). Hyperactivity/Impulsivity symptoms, on the other hand, were mainly related to Behavioral Self-Regulation, particularly involving the ability to be aware of one’s behavior, regulate emotional responses, and respond appropriately to novel or challenging situations. Overall, both parents and teachers identified consistent associations between inattentive symptoms and a wide range of EF difficulties, in line with previous findings [21,22]. These results support the robustness of the observed patterns and highlight the cross-contextual relevance of EF difficulties in children with elevated inattention symptoms.

This detailed analysis confirms the pattern already observed in the total scores, supporting the strong and cross-contextual association between ADHD symptoms—particularly inattention—and EF difficulties as perceived by both parents and teachers. However, important questions remain regarding the underlying reasons for the consistently strong associations found in the literature when using questionnaire-based tools, as opposed to performance-based neuropsychological tests [31,32]. Are these associations driven by a true conceptual overlap between the constructs measured by ADHD symptom rating scales (Conners-3) and EF impairment scales (EFQU)? Could the similarity in item phrasing lead to correlations that arise more from linguistic than from distinct but related psychological mechanisms? Or are ADHD symptoms and EF difficulties, while theoretically distinct, so tightly interconnected in everyday functioning that they become indistinguishable in informant reports?

Factor analyses were conducted to test the first hypothesis, that is, to explore whether Conners 3 and the EFQU assess the same underlying constructs, potentially explaining the high correlations as a result of overlapping item content. EFAs were first performed, yielding coherent solutions that aligned well with the theoretical structure of both instruments. Minor modifications were applied based on conceptual consistency. Subsequently, CFAs conducted on both parent and teacher reports consistently demonstrated that Conners 3 and the EFQU assess distinct and independent latent constructs. Specifically, for parent ratings, eight latent factors emerged:

1. Cognitive Self-Regulation, consisting exclusively of eleven EFQU items: ten from the Cognitive Self-Regulation subscale and one from Initiative.
2. Hyperactivity, comprising eight items from the Conners—H/I subscale.
3. Adaptability, including six EFQU items: four from the Adaptability subscale and two from Initiative.
4. Material Management, a hybrid factor composed of five EFQU items (four from the Material Management subscale and one from Cognitive Self-Regulation) and one Conners—IN item, related to the loss of belongings.
5. Inattention, entirely composed of ten Conners items: nine from the IN subscale and one from H/I.
6. Interpersonal Self-Control, grouping four EFQU items: three from Behavioral Self-Control and one from Initiative.
7. Impulsivity, a mixed factor including one EFQU item from Behavioral Self-Control and three Conners—H/I items.
8. Emotional Control, consisting of four EFQU items from the Behavioral Self-Control subscale.

All identified factors were composed exclusively of items from either the EFQU or the Conners scales, except for two mixed factors—Impulsivity and Material Management. This clear separation between the two instruments suggests that there is no substantial overlap in item phrasing across measures; if such overlap were present, one would expect more frequent cross-loadings between EFQU and Conners items. A closer inspection of the two exceptions reveals distinct patterns: the Impulsivity factor includes two items that refer almost identically to waiting for one’s turn—EFQU-P 9 and Conners-P 61—Indicating a likely overlap in phrasing rather than in conceptual content. In contrast, the Material Management factor includes items with different wording but similar meaning, such as maintaining order (EFQU-P 5 and EFQU-P 31) and losing belongings (Conners-P 97), pointing to a meaningful conceptual convergence related to the child’s ability to manage personal materials and stay organized.

For teacher ratings, five latent factors emerged:

1. Cognitive Self-Regulation, consisting exclusively of EFQU items—thirteen from the Cognitive Self-Regulation subscale and two from Self-Control.
2. Hyperactivity/Impulsivity, composed of all eleven items from the Conners—H/I subscale.
3. Inattention, entirely made up of ten items from the Conners—IN subscale.
4. Material Management, including all four EFQU items from the corresponding subscale.
5. Self-Control, comprising twelve EFQU items from the Self-Control subscale.

Notably, all five factors were composed exclusively of items from either the EFQU or the Conners scales, without any cross-loading. This structural separation highlights that, for teacher ratings, the constructs measured by the two instruments are distinct not only in content, but also in linguistic formulation; there is no evidence of overlap due to similar item phrasing. Additionally, compared to parent ratings, teacher ratings yielded a more compact factor structure, with items loading onto a smaller number of dimensions. For example, while Hyperactivity and Impulsivity emerged as distinct factors in the parent data, they loaded onto a single unified factor in the teacher model.

All subscales demonstrated acceptable-to-excellent internal consistency for parent ratings and excellent internal consistency for teacher ratings, indicating that the items within each factor reliably reflect a coherent and distinct underlying construct. This strong internal alignment reinforces the theoretical soundness of the models and suggests that the EFQU and the Conners 3 questionnaires capture separable dimensions of functioning. Despite the associations observed, the structure and internal coherence of each scale support the idea that EF difficulties and ADHD symptoms, as measured by these tools, are conceptually and empirically distinct. Accordingly, the first two hypotheses—suggesting that correlations may result from similar item content or phrasing—can be reasonably rejected for both parent and teacher ratings.

Taken together, these results support the third and final hypothesis: although EF difficulties and ADHD symptoms are theoretically distinct constructs, they are empirically interrelated in everyday functioning, as reported by parents and teachers. The presence of significant correlations among latent factors—particularly between Cognitive Self-Regulation and Inattention—likely reflects the functional interplay between these domains in daily behavior, rather than the existence of a shared underlying construct. If a higher-order factor were present, more frequent cross-loadings and less clearly differentiated factor structures would be expected, which was not observed in the current models. The strong and consistent associations observed across both parent and teacher ratings reinforce this interpretation, aligning with the previous literature that has stressed the tight interplay between EF and ADHD symptomatology (e.g., [21,22]).

Notably, the pattern of associations differed between parent and teacher reports. For parents, correlations tended to occur primarily between factors originating from the same questionnaire. For instance, Adaptive Flexibility, Interpersonal Self-Control, Emotional Control (composed exclusively of EFQU items), and Material Management (composed predominantly of EFQU items) were mainly associated with other EFQU-based factors. Likewise, Conners-based factors such as Hyperactivity and Impulsivity were correlated mainly with other Conners-derived dimensions. The only notable exception was the strong link between Cognitive Self-Regulation (EFQU-composed) and Inattention (Conners-composed), both of which also showed associations within their own respective domains. This pattern suggests that parents may perceive EF difficulties and ADHD symptoms as related but distinct dimensions, reflecting a more analytical perspective of children’s functioning.

In contrast, teacher ratings revealed a more integrated profile. Most factors—particularly Inattention, Material Management, and Self-Control—were significantly associated with nearly all other domains, regardless of their source questionnaire. The only exception was the absence of a significant correlation between Cognitive Self-Regulation and Hyperactivity/Impulsivity. This broader pattern of associations suggests that teachers may observe EF and behavioral symptoms as part of a unified, global picture of classroom functioning, where regulatory and attentional difficulties tend to co-occur and influence one another. Interestingly, both informants identified a particularly strong association between Cognitive Self-Regulation and Inattention, reinforcing the centrality of these dimensions in the ADHD–EF link [21].

These findings have important implications for clinical practice; for instance, they highlight the importance of using multiple informants to capture the full spectrum of executive and behavioral functioning in children, as parents and teachers may contribute complementary perspectives [6]. Parents often observe behavior in more differentiated and context-specific settings, typically within one-to-one or small group interactions at home. In contrast, teachers observe children in broader, more dynamic social contexts, which may foster a more holistic and situationally integrated view of functioning [7]. Moreover, these differences may also be attributed to the distinct behaviors that children display at home versus at school [8]. This discrepancy in observational context does not imply that one rater is more reliable than the other, but rather reflects the specific contexts and relationships in which behavior is observed, and the complementary nature of these perspectives. Such data highlight the importance of recognizing contextual variability as clinically relevant information, encouraging a shift away from models that view children’s functioning as consistent and uniform across settings and situations. For this reason, it is recommended to involve multiple informants in the assessment process in order to obtain a more accurate and context-sensitive understanding of the child’s functioning. Lastly, this comprehensive approach may improve the accuracy of diagnostic formulations and inform more tailored intervention strategies by allowing for the development of context-specific interventions that address the child’s unique needs across different settings.

## 5. Conclusions

Previous studies have shown that EF difficulties and ADHD symptoms, when assessed via rating scales, tend to be closely associated and often co-occur in everyday functioning. Building on this evidence, the present study aimed not only to confirm this empirical association, but also to clarify the underlying reasons for it, by progressively ruling out alternative explanations. Using the Conners 3 Rating Scales to assess ADHD symptoms and the EFQU to assess EF impairments, data were collected from a large sample of 1068 children aged 7–14 years, based on both parent and teacher ratings. As a first step, the expected pattern of strong correlations was replicated: both informants reported significant associations, particularly between inattentive symptoms and a wide range of EF difficulties. Subsequently, exploratory and confirmatory factor analyses were conducted to test whether these associations might be due to overlap in item phrasing or construct similarity. Findings showed that the Conners 3 and EFQU capture distinct latent dimensions of functioning, with virtually no overlap in item content. Specifically, in the parent ratings, eight latent factors emerged, six of which were composed exclusively of items from either the EFQU or Conners, and two hybrid factors where overlapping construct domains or phrasing may have played a role. In contrast, teacher ratings yielded five well-separated, “clean” factors, further supporting the structural distinctiveness of the two instruments. In a final step, the associations between the extracted latent factors were analyzed. The strength and consistency of these associations reinforce the idea that, although ADHD symptoms and EF impairments are conceptually distinct, they are empirically intertwined in children’s day-to-day behavior. This convergence was consistently observed across both parent and teacher reports. Interestingly, the pattern of associations differed slightly by informant: teachers tended to provide a more global, integrated view of functioning—likely influenced by their experience observing children in dynamic, multi-tasking environments—whereas parents appeared to perceive ADHD symptoms and EF difficulties as more analytically distinct, possibly reflecting their one-on-one interactions at home. Taken together, these findings have important implications for clinical practice, highlighting the importance of relying on multi-informant perspectives in the clinical and educational assessment of children. They also underscore the value of considering contextual variability when interpreting questionnaire data, as children may behave differently across settings and relationships. Ultimately, understanding how ADHD symptoms and EF difficulties converge and diverge across tools and contexts can improve the accuracy of diagnostic formulations and guide more tailored intervention strategies.

### Limitations of the Study and Future Directions

This study presents several strengths, including a large sample size, the use of two well-validated rating instruments completed by both parents and teachers, and a rigorous, data-driven statistical approach. The adoption of a multi-informant design enhances the ecological validity of the findings, while the dimensional framework provides a nuanced understanding of the relationship between executive functioning and ADHD-related traits. However, some limitations must be acknowledged. First, the presence of missing data may have influenced the overall robustness of the analyses. Nevertheless, missingness was handled using appropriate techniques, ensuring internal consistency across comparisons.

Second, the study focused exclusively on rating scales and did not include performance-based measures of executive function. While this choice aligns with the study’s primary aim—investigating the basis of correlations commonly observed between questionnaires—it limits the ability to draw conclusions about the convergence or divergence between informant-based and task-based assessments. Moreover, the study relied on only one rating scale for ADHD symptoms and one for EF abilities; using multiple subjective measures for each construct would increase the specificity and sensitivity of the scores produced. Future studies should extend this work by including neuropsychological tests and a broader range of questionnaires to examine whether similar or divergent associations emerge across methodologies.

Third, the sample was not clinically diagnosed for ADHD. However, this limitation is partly mitigated by the nature of the analyses: correlational and factor-based approaches are not dependent on clinical thresholds, and focusing on symptom dimensions allows for a nuanced, dimensional understanding of the relationship between ADHD traits and EF difficulties. Additionally, recent research supports a dimensional conceptualization of ADHD, viewing it as a spectrum of attentional and behavioral traits rather than a unitary disorder [43].

Fourth, although the sample covers a broad age range (7–14 years), the results may not be generalizable to younger children (e.g., preschoolers), for whom both ADHD symptoms and executive functioning abilities may present differently. In early childhood, ADHD symptoms are often more externally visible and behaviorally driven, while executive functions are still in the early developmental stages. By focusing on school-aged children, the present study ensured greater internal consistency in behavior patterns and developmental expectations. Moreover, the lower representation of children at the younger and older ends of the age range may limit the generalizability of the findings for those subgroups. Additionally, information on socioeconomic status (SES) was not systematically collected, limiting our ability to examine its potential moderating effects on the observed associations.

Fifth, the timing of questionnaire completion was not systematically recorded, and it showed considerable variability across participants. Although the estimated completion time for each questionnaire was brief (20–30 min), the period between questionnaire delivery and return ranged from days to several weeks or even months. Future studies should consider monitoring or standardizing the timing of completion to improve the temporal validity of the data collected.

Future research could expand on these findings by investigating whether the same patterns of association and dissociation hold when comparing questionnaire-based assessments to performance-based tests. Such studies could clarify whether the empirical overlap observed between rating scales is rooted in shared real-world behaviors, shared method variance, or both. Moreover, future work might explore whether similar factor structures emerge in younger or older populations and how these associations evolve developmentally through longitudinal designs, or how cultural and contextual factors shape informant perceptions. Finally, given the conceptual ambiguity of executive functioning in the literature, future studies may benefit from comparing alternative theoretical and statistical models to examine whether different operationalizations of EF yield consistent patterns of association.

## Figures and Tables

**Table 1:** Distribution of participants by age and gender.

	Age (Years)							
	7	8	9	10	11	12	13	14
Male	3	52	86	95	104	71	74	43
Female	6	43	98	111	85	69	60	29
Total	9	95	184	206	189	140	134	72

**Table 2:** Descriptive statistics, skewness, kurtosis, and missing data for all subscales.

	Mean	SD	Range	Skewness	Kurtosis	Missing Data (%)
Conners–IN (Parent)	52.12	11.33	33–95	0.85	0.63	34.0
Conners–H/I (Parent)	52.04	12.01	32–109	1.23	1.80	34.0
Conners–IN (Teacher)	52.23	14.15	33–137	2.08	6.61	21.6
Conners–H/I (Teacher)	53.08	18.06	38–254	4.97	37.24	21.6
EFQU-P—Cognitive Self-Regulation	42.65	8.82	15–55	−0.62	−0.23	24.1
EFQU-P—Behavioral Self-Control	34.21	6.14	10–45	−0.56	0.21	24.1
EFQU-P—Material Management	13.54	3.92	4–20	−0.26	−0.56	24.1
EFQU-P—Adaptability	15.06	3.20	4–20	−0.57	0.12	24.1
EFQU-P—Initiative	15.89	2.73	5–20	−0.55	0.16	24.1
EFQU-P—Total EF score	121.35	20.33	61–160	−0.38	−0.22	24.1
EFQU-T—Self-Regulation	55.04	12.14	14–70	−0.83	0.22	6.9
EFQU-T—Self-Organization	52.99	12.82	14–70	−0.55	−0.40	6.9
EFQU-T—Material Management	15.84	4.17	4–20	−1.01	0.35	6.9
EFQU-T—Total EF score	123.88	26.63	34–160	−0.67	−0.12	6.9

**Table 3:** Cronbach’s alpha coefficients for CFA-derived latent factors in parent ratings.

Factor	Cronbach’s α
1. Cognitive Self-Regulation	α = 0.94
2. Hyperactivity	α = 0.83
3. Adaptability	α = 0.82
4. Material Management	α = 0.89
5. Inattention	α = 0.88
6. Interpersonal Self-Control	α = 0.75
7. Impulsivity	α = 0.72
8. Emotional Control	α = 0.71

**Table 4:** Cronbach’s alpha coefficients for CFA-derived latent factors in teacher ratings.

Factor	Cronbach’s α
1. Cognitive Self-Regulation	α = 0.97
2. Impulsivity/Hyperactivity	α = 0.94
3. Inattention	α = 0.95
4. Material Management	α = 0.95
5. Self-Control	α = 0.97

**Table 5:** Significant Spearman correlations ≥ 0.50 among parent-rated factors.

	1	2	3	4	5	6	7	8
1. Cognitive Self-Regulation	-		0.54 **	0.63 **	−0.70 **	0.64 **		0.51 **
2. Hyperactivity		-			0.63 **		0.60 **	
3. Adaptive Flexibility	0.54 **		-			0.53 **		0.54 **
4. Material Management	0.63 **			-				
5. Inattention	−0.70 **	0.63 **			-		0.50 **	
6. Interpersonal Self-Control	0.64 **		0.53 **			-		0.66 **
7. Impulsivity		0.60 **			0.50 **		-	
8. Emotional Control	0.51 **		0.54 **			0.66 **		-

Note. Spearman’s rho coefficients. N = 604. ** = *p* < 0.001.

**Table 6:** Significant Spearman correlations ≥ 0.50 among teacher-rated factors.

	1	2	3	4	5
1. Cognitive Self-Regulation	-		−0.79 **	−0.73 **	−0.71 **
2. Impulsivity/Hyperactivity		-	−0.61 **	−0.58 **	−0.70 **
3. Inattention	−0.79 **	−0.61 **	-	−0.66 **	−0.63 **
4. Material Management	−0.73 **	−0.58 **	−0.66 **	-	0.76 **
5. Self-Control	−0.71 **	−0.70 **	−0.63 **	0.76 **	-

Note. Spearman’s rho coefficients. N = 778. ** = *p* < 0.001.

## Data Availability

The data presented in this study are available on request from the corresponding authors. The data are not publicly available due to privacy restrictions.

## Abbreviations

The following abbreviations are used in this manuscript:

ADHD	Attention-Deficit/Hyperactivity Disorder
EF	Executive Function
EFQU	Executive Function Questionnaire
WM	Working Memory
CFA	Confirmatory Factor Analysis
EFA	Exploratory Factor Analysis
KMO	Kaiser–Meyer–Olkin
RMSEA	Root Mean Square Error of Approximation
CFI	Comparative Fit Index
TLI	Tucker–Lewis Index
SRMR	Standardized Root Mean Square Residual
IN	Inattention
H/I	Hyperactivity/Impulsivity
DSM-5	Diagnostic and Statistical Manual of Mental Disorders, 5th Edition
SPSS	Statistical Package for the Social Sciences
IL	Illinois
SES	Socioeconomic Status
